# PD-L1 expression in urothelial bladder cancer varies more among specimen types than between companion assays

**DOI:** 10.1007/s00428-021-03094-6

**Published:** 2021-04-28

**Authors:** Joep J. de Jong, Hans Stoop, Joost L. Boormans, Geert J.L.H. van Leenders

**Affiliations:** 1 Department of Urology, Erasmus MC University Medical Centre, Rotterdam, The Netherlands; 2 Department of Pathology, Erasmus MC University Medical Centre, Rotterdam, The Netherlands

**Keywords:** Urothelial cancer, Bladder, PD-L1, Immunohistochemistry, Concordance

## Abstract

Urothelial bladder cancer (UBC) patients ineligible to platinum-based chemotherapy can be treated with immune-checkpoint inhibitors (ICI) in Programmed Death Ligand 1 (PD-L1) positive cases. Although concordance exists between different PD-L1 assays, little is known on PD-L1 expression variability in matched UBC samples. We compared PD-L1 expression in whole slides of matched transurethral resections (TURBT), radical cystectomies (RC), and lymph node metastasis (LN). Immunohistochemistry using the VENTANA PD-L1 (SP263) assay was performed on 115 patients and scored positive if expression occurred in ≥25% immune cells (IC), ≥25% tumour cells (TC), or both. PD-L1 was positive in 42.7% TURBT, 39.8% RC, and 27.3% LN specimens. Concordance was moderate (κ=0.52; *P*<0.001) between TURBT and RC, and fair between LN and TURBT (κ=0.31; *P*=0.048) or RC (κ=0.25; *P*=0.075). Comparison with the VENTANA PD-L1 (SP142) assay which had been performed previously on the same cohort showed moderate to substantial inter-assay agreement (κ=0.42–0.66). Although TC staining is not part of the SP142 scoring algorithm, discordant PD-L1 assay outcome could be attributed to SP263 TC≥25% staining in only 41% of cases. These results show that PD-L1 expression variability between matched specimens is higher than that between individual assays. Optimal specimen determination for PD-L1 testing needs to be addressed in future studies.

**Supplementary Information:**

The online version contains supplementary material available at 10.1007/s00428-021-03094-6.

## Introduction

With a global annual incidence of 430,000 patients, bladder cancer is the fourth and tenth most common cancer in men and women, respectively [1]. From these patients, approximately 25% present with muscle-invasive bladder cancer (MIBC). According to American Urological Association (AUA) guidelines, neo-adjuvant cisplatin-based chemotherapy (NAC) followed by radical cystectomy (RC) is the recommended treatment for MIBC [2]. Despite this aggressive treatment regimen, the 5-year overall survival of MIBC patients is only 55%. Importantly, the overall incidence and mortality rate have undergone little change in the past decades.

Many immunotherapeutic agents targeting Programmed cell Death 1 (PD-1) receptor and its PD-L1 ligand are currently tested in clinical trials, offering new opportunities for the treatment of advanced urothelial cancer patients [3]. In recent studies, higher response rates were observed in patients with high expression of PD-L1 in tumour tissue. Consequently, first-line use of atezolizumab and pembrolizumab for patients being ineligible to cisplatin-based chemotherapy has been restricted to PD-L1 positive tumours [4].

Although immunohistochemical PD-L1 expression may serve as a measure for effectiveness of immune-checkpoint inhibitors (ICI), there are some studies that did not show significant predictive value among PD-L1 subgroups [5, 6]. Furthermore, the use of different PD-L1 companion diagnostic tests, scoring algorithms and cut-off points have raised the question on how to implement immunohistochemical assays in clinical practice. Multiple studies have shown overall good concordance between different PD-L1 assays [7–9]. However, little is known about the variability of PD-L1 expression among different tumour tissues from individual patients [10, 11]. Previously, we found poor concordance of PD-L1 expression in urothelial cancer as determined in matched transurethral resection of the bladder tumour (TURBT), RC, and lymph node metastasis (LN) using the VENTANA PD-L1 (SP142) assay [12]. The PD-L1 (SP142) assay has been used as companion diagnostic for atezolizumab and is based on PD-L1 expression on tumour-associated immune cells (IC) only. Since IC reflect an inflammatory reaction to genomically aberrant tumour cells (TC), but are not considered genetically changed themselves, we hypothesise that a PD-L1 assay taking TC expression into account would be more stable among matched urothelial cancer specimens. The VENTANA PD-L1 (SP263) assay, which is used as companion diagnostic for durvalumab, takes into account PD-L1 expression on both TC and IC, and has, like the SP142 assay, been developed for a VENTANA BenchMark ULTRA platform. The objective of this study was to determine the concordance of PD-L1 expression using the SP263 assay in matched TURBT, RC, and LN samples, and to compare its outcome with the SP142 assay, which had been performed previously on the same population [12].

## Materials and methods

### Patient selection and pathological review

In total, we included 115 patients who underwent RC with bilateral pelvic lymph node dissection (PLND) for cT2-T4aN0N1M0 viable urothelial carcinoma of the bladder, at the Erasmus MC University Medical Centre, Rotterdam, The Netherlands, between 1998 and 2017. Thirty-five patients had received (neo)adjuvant therapy before operation. We selected cases for the availability of matched TURBT, RC, and/or LN specimens, as we aimed to investigate PD-L1 assay performance in matched primary bladder cancer and metastases. The use of patient material for scientific purposes was approved by the local Medical Research Ethics Committee (Rotterdam, Netherlands, MEC-2014-553). All haematoxylin and eosin (HE) slides were reviewed by a genitourinary pathologist (GvL), who monitored the following: grade (WHO1973 and 2016), pT stage (TNM 8th edition), surgical margin status, presence of carcinoma in situ (CIS), vascular invasion (VI), and presence of variant histology (squamous, glandular, neuroendocrine, sarcomatoid) [12]. In the present study, whole tissue slides were newly stained with the PD-L1 SP263 assay and compared to PD-L1 SP142 stainings, which had been performed on the same slides and published previously [12].

### Immunohistochemistry and scoring

**Fig. 1:**
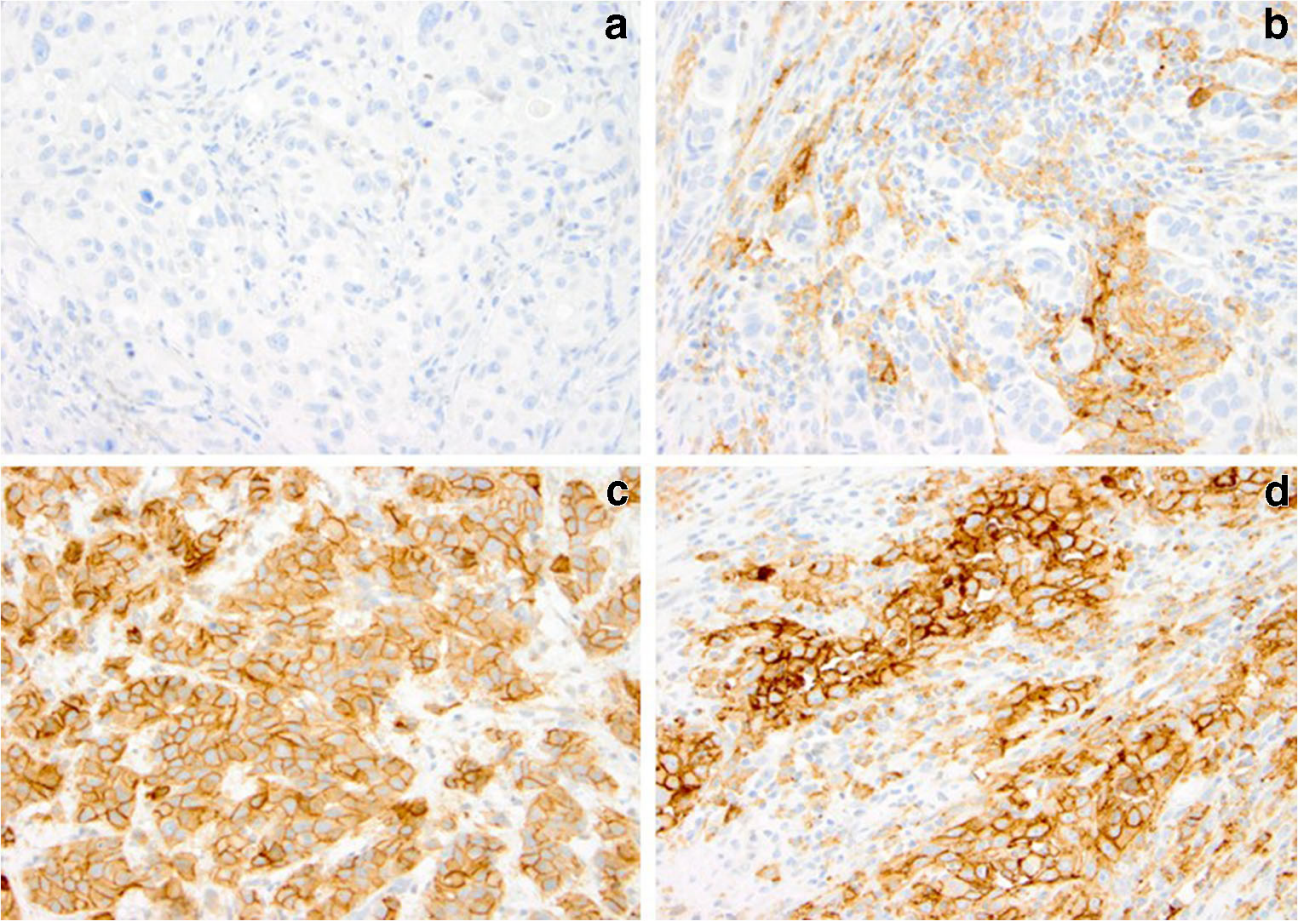
PD-L1 staining in urothelial carcinoma of the urinary bladder. According to the manufacturer’s guidelines, the SP263 assay is positive if PD-L1 expression occurs in ≥25% of either immune cells (IC) or tumour cells (TC) (IC≥25% and/or TC≥25%). a PD-L1 negative: limited expression in immune cells; tumour cells negative (IC 5–10%; TC 0%). b PD-L1 positive: strong expression in immune cells; tumour cells negative (IC 70%; TC 0%). c PD-L1 positive: strong expression in tumour cells; immune cells negative (IC 0%; TC 100%). d PD-L1 positive: strong expression in both immune and tumour cells (IC 70%; TC 100%). PD-L1 (SP263 assay), original magnifications × 20

Four-micron consecutive sections were cut from representative formalin-fixed, paraffin-embedded (FFPE) diagnostic tissue blocks, mounted on adhesive glass slides and stained for PD-L1 using the SP263 and SP142 assays on the VENTANA BenchMark ULTRA platform, according to the manufacturer’s protocols (Ventana Medical Systems, Tucson, AZ, USA) [6, 13–16]. For both assays, PD-L1 staining on IC, including lymphocytes, macrophages, histiocytes, reticular dendritic cells, plasma cells, and neutrophils, was scored within the tumour reactive stroma, between the tumour islands and invading the tumour border. Samples stained with SP142 are considered positive if PD-L1 expression in IC covers ≥5% of the tumour area (IC≥5%). Samples stained with SP263 are positive if PD-L1 expression occurs in ≥25% of either IC or TC (IC≥25% and/or TC≥25%) (Fig. 1). Full details of the VENTANA SP263 and SP142 PD-L1 assay evaluation and scoring algorithms are provided in the manufacturer’s manuals [15, 16]. PD-L1 expression was scored by one pathologist (GvL) with experience in PD-L1 assay assessment [17]. PD-L1 staining with the SP142 assay had been performed on consecutive slides of the same cohort and was used in the current study for comparison with the SP263 assay [12].

### Statistical analyses

For analysis of PD-L1 assay outcomes and clinicopathological characteristics, we used two-sided Wilcoxon rank-sum test for continuous and non-normally distributed data. Categorical data was analysed by Fisher’s exact tests. Intra- and inter-assay agreement of PD-L1 expression in matched TURBT, RC, and LN specimens was evaluated using Cohen’s κ coefficients. These coefficients were used to score the concordance as follows: ‘no agreement’ (κ<0), ‘slight’ (κ=0–0.20), ‘fair’ (κ=0.21–0.40), ‘moderate’ (κ=0.41–0.60), ‘substantial’ (κ=0.61–0.80), or ‘almost perfect’ (κ=0.81–1). We constructed modified Venn diagrams to visualise inter-assay concordance of SP142 and SP263 assays. *P*-values <0.05 were considered significant. All statistical analyses were conducted using SPSS software (version 25) and R version 3.4.4.

## Results

### Patient characteristics

The clinicopathological patient characteristics at time of RC of the 115 patients are summarised in Table 1. Median patient age at time of RC was 65.7 years (interquartile range (IQR) 57.9–72.3 years). All patients had undergone TURBT for MIBC (≥pT2). In total 109/115 (94.8%) patients had undergone cystectomy; in 6 patients cystectomy was omitted because of intra-operative identification of lymph node metastasis. Thirty-five (30.4%) patients had received pre-operative neo-adjuvant therapy, including chemotherapy (*n*=27), radiation (*n*=6), or chemoradiation (*n*=2). PLND was performed in 109 (94.8%) patients of whom 57 (52.3%) had lymph node metastasis. Perivesical lymph nodes (PVLN) were identified in 32 patients and were positive in 11 (34.4%) cases. In 3 patients, metastases were present in perivesical but not pelvic lymph nodes, resulting in a total of 60 (55%) patients with metastatic disease at time of operation.

**Table 1:** Clinicopathological characteristics of the cohort at time of radical cystectomy

Parameter	Number
Gender	
Male Female	90 25
Neo-adjuvant therapy	
No Chemotherapy Radiation Chemoradiation	80 27 6 2
Type cystectomy	
Cystoprostatectomy Simple cystectomy Partial cystectomy Resection bladder, uterus, vagina No cystectomy performed	79 7 5 18 6
Histology	
Pure urothelial carcinoma (UC) UC with variant histology No invasive tumour (pT0,pTa,ypT0, ypTis) Unknown No cystectomy performed	54 42 7 6 6
(Concomitant) carcinoma in situ (CIS)	
Present Absent Unknown No cystectomy performed	38 69 2 6
Combined Grade UC (WHO 1973 & 2016)	
T0/Ta/Tis Grade 2 (LG) Grade 2 (HG) Grade 3 (HG) No cystectomy performed	7 1 10 91 6
pT stage	
pT0 pTis pTa pT1 pT2 pT3 pT4 No cystectomy performed	4 2 1 11 24 52 15 6
Surgical margin status	
Negative Positive T0/Ta/Tis No cystectomy performed	90 12 7 6

### PD-L1 (SP263) expression

The SP263 assay was positive in 38/89 (42.7%) TURBT, 39/98 (39.8%) RC, and 12/44 (27.3%) LN specimens. PD-L1 expression using the SP263 assay is considered positive based on its staining in IC, TC, or both (Fig. 1). PD-L1 expression was present in ≥25% of IC only in 19/89 (21.3%), 16/98 (16.3%), and 7/44 (15.9%) of TURBT, RC, and LN specimens; in ≥25% TC only in 14/89 (15.7%), 15/98 (15.3%), and 4/44 (9.1%); and in both IC and TC ≥25% in 5/89 (5.6%), 8/98 (8.2%), and 1/44 (2.2%), respectively (Table 2).

**Table 2:** PD-L1 expression score of the SP263 assay

Tissue type	SP 263 assay score			
	IC or TC ≥25%	IC ≥ 25%	TC ≥ 25%	Both IC and TC ≥25%
TURBT				
Yes No	38 (42.7%) 51 (57.3%)	19 (21.3%) 70 (78.7%)	14 (15.7%) 57 (84.3%)	5 (5.6%) 84 (94.4%)
Total	89	89	89	89
Cystectomy				
Yes No	39 (39.8%) 59 (60.2%)	16 (16.3%) 82 (83.7%)	15 (15.3%) 83 (84.7%)	8 (8.2%) 90 (91.8%)
Total	98	98	98	98
LN+				
Positive negative	12 (27.3%) 32 (72.7%)	7 (15.9%) 37 (84.1%)	4 (9.1%) 40 (90.1%)	1 (2.2%) 43 (97.7%)
Total	44	44	44	44

### PD-L1 (SP263) in variant histology

Variant histology was observed in 42 RC specimens, while 54 had pure invasive urothelial carcinoma, and no information on variant histology was available in 6 cases. The following variant histologies were observed: squamous (*n*=16), glandular (*n*=14), micropapillary (*n*=5), sarcomatoid (*n*=5), diffuse (*n*=7). Ninety-three RC cases had evaluable SP263 staining and information on the presence of variant histology in the entire specimen, of which 41 had variant histology. The SP263 assay was positive in 16/41 (39.0%) cases with and in 18/52 (34.6%) without variant histology (*P*=0.67). PD-L1 was positive in 8/16 (50.0%) cases with squamous differentiation and in 26/77 (33.8%) without (*P*=0.26). Of the 13 RC specimens with aberrant glandular differentiation, 3 (23.1%) were PD-L1 positive against 31/80 (38.8%) without glandular differentiation (*P*=0.36). The frequency of micropapillary, sarcomatoid, and diffuse histology was too low for statistical analysis.

### PD-L1 (SP263) concordance in matched TURBT and RC specimens

**Fig. 2:**
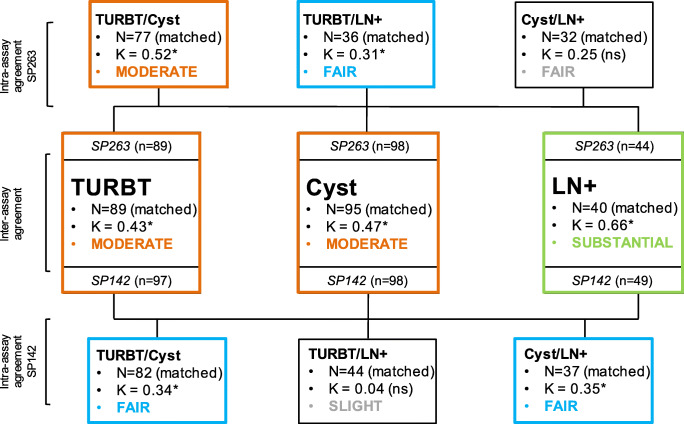
Intra- and inter-assay agreement of PD-L1 expression for matched TURBT, cystectomy, and LN+ specimens

Fifty-nine of the 77 (76.6%) patients with matched TURBT and cystectomy specimens had concordant PD-L1 expression status, being positive in 23 and negative in 36 cases (**Fig.** **2**, **Table S****1**). Eight patients had positive PD-L1 status in TURBT but not in subsequent RC specimens, and 10 showed PD-L1 expression in RC specimens only (κ=0.52, *P*<0.001; **Fig.** **2**, **Table S****2**). Six out of 18 (33.3%) patients with discordant assay outcome had received neo-adjuvant therapy, compared to 15/59 (25.4%) of concordant cases (*P*=0.55). Eight out of 18 (44.4%) cases with IC≥25% at TURBT also had IC≥25% at RC, while 10/18 (55.6%) had not. 15/17 (88.2%) tumours with TC≥25% at TURBT were TC PD-L1 positive on RC. On the other hand, 8/19 (42.1%) patients with IC≥25% on RC were IC PD-L1 positive at the preceding TURBT specimen as compared to 15 out of 20 (75.0%) RC cases with TC≥25% (**Table** 3**A**).

**Table 3:**
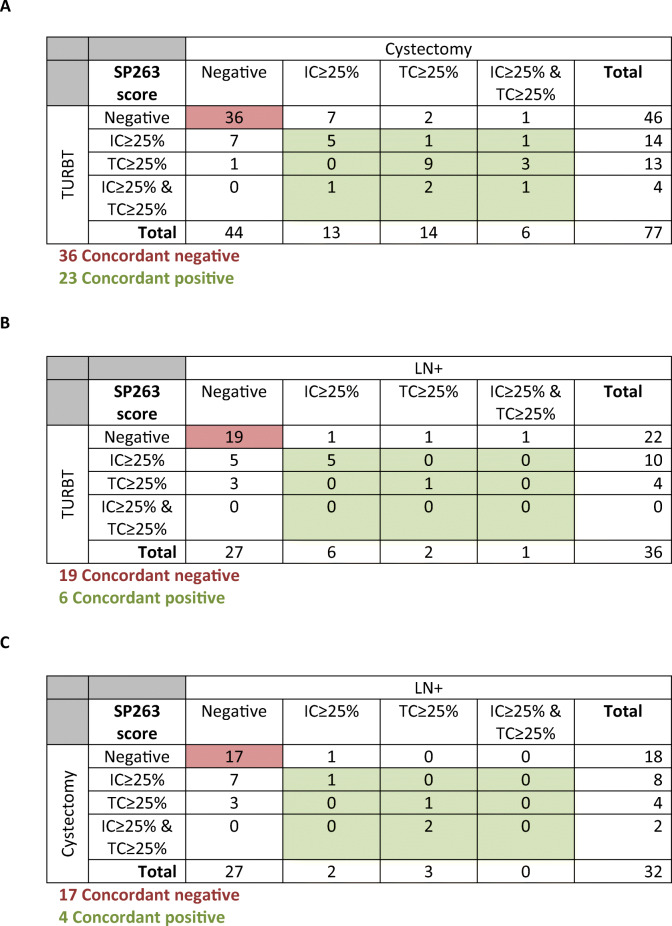
Intra-assay agreement of the SP263 assay

### PD-L1 (SP263) concordance in matched TURBT and LN specimens

Matched TURBT and LN samples had concordant PD-L1 status in 25/36 (69.4%) cases, being positive in 6 and negative in 19 subjects (**Fig.** **2****, Table S****2**). Eight out of 11 discordant cases showed PD-L1 expression in the TURBT specimen only, and 3 in the LN metastasis (κ=0.31, *P*=0.048; **Fig.** **2**, **Table S****2**). We observed that among the 6 concordant positive cases, the SP263 assay was scored as being positive for its expression on [1] IC≥25% for both TURBT and LN in 5/6 (83.3%) cases, and [2] TC≥25% for both TURBT and LN in 1/6 (16.7%) cases (**Table** <b>3</b>**B**). Of the 11 (30.6%) discordant cases, either specimen was scored positive for PD-L1 expression in IC (6/11; 54.5%), TC (4/11; 36.4%), or both (1/11; 9.1%).

### PD-L1 (SP263) concordance in matched RC and LN specimens

Twenty-one of 32 (65.6%) patients had concordant SP263 assay outcome in matched RC and LN samples, of whom 4 were positive and 17 negative (**Fig.** **2****, Table S****2**). Of the 11 discordant cases, 10 were PD-L1 positive and one was negative at RC (κ=0.25, *P*=0.075; **Fig.** **2**, **Table S****2**). We observed that among 4 concordant positive cases, the SP263 assay was scored positive by its staining of [1] IC≥25% for both RC and LN in 1/4 cases and [2] TC≥25% for both RC and LN in 1/4 cases, and [3] in 2/4 concordant cases, the LN metastasis had TC≥25%, while RC had both IC and TC≥25% (**Table** <b>3</b>**C**). Of the 11 discordant cases, the RC or LN specimen was positive based on PD-L1 expression in IC (8/11; 72.7%) or TC (3/11; 27.3%).

### PD-L1 SP263 and SP142 inter-assay comparison

**Fig. 3:**
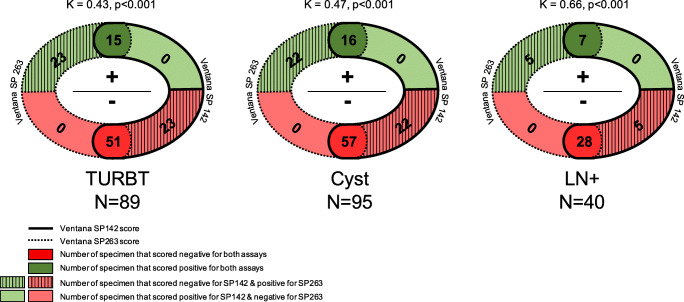
Modified Venn diagrams for the inter-assay agreement of both PD-L1 assay scores in matched TURBT, cystectomy, and LN+ specimens

PD-L1 expression had previously been scored on consecutive sections of the same paraffin blocks using the SP142 assay [12]. The SP263 assay led to more frequent positive PD-L1 expression status than the SP142 assay in TURBT (42.7% versus 15.5%; *P*<0.001), RC (39.8% versus 17.3%; *P*<0.001), and LN specimens (27.3% versus 18.4%; *P*=0.33) (**Table S****1**). Inter-assay agreement was moderate in TURBT (κ=0.43, *P*<0.001) and RC (κ=0.47, *P*<0.001), and substantial (κ=0.66, *P*<0.001) in LN specimens (**Fig.** **3****, Table S****2**). All discordant cases were positive for PD-L1 expression by SP263 while being negative by SP142. Since the SP263 but not the SP142 assay takes PD-L1 expression in TC into account, we hypothesised that discordance in assay outcome might be explained by PD-L1 expression in urothelial carcinoma cells. However, we observed that among discordant cases, SP263 was scored positive based on PD-L1 expression in TC≥25% only in 7/23 (30.4%) TURBT, 11/22 (50%) RC, and 0/4 (0%) LN specimens (**Table S****3**). Considering all 44 discordant cases with ≥25% IC or TC staining in the three sample types using the SP263 assay, 18 (40.9%) were discordant because of SP263 TC≥25% staining. Finally, the median specimens’ age was not significantly associated with assay discordance excluding more rapid degeneration of the SP142 assay epitope (**Figure S****1**).

## Discussion

Since PD-L1 testing is required for starting first-line treatment with atezolizumab and pembrolizumab in urothelial carcinoma, it is important to elucidate what assay and specimen type are most representative for prediction of therapeutic response. While several studies have indicated overall inter-assay concordance rates of 59–93% [7], little is known yet on PD-L1 variability among matched tumour specimens [10, 11]. In this study, we found that PD-L1 status using the SP263 assay on whole tissue sections showed moderate agreement (κ=0.52) between TURBT and RC specimens, and fair agreement (κ=0.25–0.31) between both specimens and LN metastasis. In RC specimens, PD-L1 expression in TC was more stable than in IC, as 75% and 50% of RC specimens with TC≥25% also had positive TC PD-L1 status in TURBT and LN, respectively, as compared to 47% and 10% of cases with IC≥25%. Furthermore, use of the SP263 assay resulted in more frequent positive PD-L1 status than the SP142 assay, with moderate to substantial inter-assay agreement (κ=0.42–0.66). Although TC staining is not part of the SP142 scoring algorithm, discordant PD-L1 assay outcome could be attributed to SP263 TC≥25% only staining in 41%. Concordance of PD-L1 (SP263) expression between specimens (κ=0.25–0.52) of the same patient was lower than between both SP263 and SP142 assays (κ=0.42–0.66). Therefore, PD-L1 expression varies more among matched specimen types than between individual assays.

While various studies have investigated the performance of different PD-L1 assays in urothelial cancer, it is not known yet what tissue specimen, sampling technique and time of sampling are most representative for determination of PD-L1 status. Clinical trials have used a broad range of archival specimens for PD-L1 immunohistochemistry, including biopsies and excisions of primary and metastatic sites, before and after (neo-)adjuvant chemotherapy. The impact of this variability has only rarely been subject to investigation. Within the second-line atezolizumab trial using SP142 as companion diagnostic, Rosenberg et al. reported that PD-L1 expression was higher in resection specimens (39%) and TURBT (34%) than in primary lesion biopsies (17%) or metastasis (8%) [13]. In the current study, we showed fair to moderate agreement (κ=0.25–0.52) of PD-L1 outcome using the SP263 assay in matched urothelial cancer specimens, which is higher than for the SP142 assay (κ=0.05–0.35), which we reported previously [12]. A low agreement rate between primary and metastatic lesions was also found for the SP142 assay by Burgess et al. (κ=0.086) [11]. The higher PD-L1 concordance rate between matched TURBT, RC, and LN specimens found for the SP263 than for the SP142 assay indicates SP263 is a more robust assay for PD-L1 assessment. At this moment, however, it is unclear what specimen type is most representative for PD-L1 assessment. Since SP263 shows less variability between specimens types of the same patient, its use is preferred over SP142 for urothelial carcinoma. Only if multiple specimens are tested for PD-L1 expression status in patients who are treated by ICI, it will become clear which specimen type is most representative for predicting response.

In this study we found moderate to substantial agreement (κ=0.43–0.66) between the SP263 and SP142 assay in whole tissue sections of urothelial bladder cancer. This is in line with the agreement (κ=0.582) observed in our previous study, in which we performed a tissue microarray (TMA)–based inter-assay concordance study of another urothelial cancer cohort [17]. Most concordance studies have been performed on TMAs, and it might be questioned to what extent these are representative for whole tissue sections, which are not used for PD-L1 expression analysis in clinical practice. Wang et al. found moderate concordance between matched TMA and whole tissue sections with slightly higher agreement for the SP263 (κ=0.573) than the SP142 (κ=0.493) assay [18]. In fact, most inter-assay agreement studies found best concordance rates between 22C3 and SP263 assay, while discrepant outcome was more frequent for SP142 [7, 8, 10, 17]. Since PD-L1 expression status is increasingly required for urothelial cancer management, it is important to develop standards for its testing. Apart from specimen type, local availability of the immunohistochemical staining platform limits the choice for PD-L1 companion assay use. Since the 22C3 and 28-8 assays have been developed and optimised for a DAKO staining platform, and the SP142 and SP263 assays for the VENTANA BenchMark ULTRA platform, companion diagnostic selection is highly dependent on the technical equipment being present at the pathology department. Due to the high concordance rate of 22C3 and SP263, which both take TC and IC staining into account, these assays might serve as first choice depending on the platform present. If a pathology laboratory has the availability of a DAKO staining platform, 22C3 is applied together with its assay-specific scoring algorithm (combined positive score (CPS), IC and TC ≥10%). In case a VENTANA BenchMark ULTRA platform is present, the SP263 assay can be applied as surrogate for 22C3 companion diagnostics. In the latter case, a relevant and yet unanswered question is whether the SP263 assay staining should be evaluated according to its manufacturer’s algorithm (IC or TC ≥25%) or the 22C3 companion algorithm (CPS>10) for pembrolizumab treatment selection. We scored the SP263 stainings also following the CPS algorithm and found substantial agreement (kappa 0.68–0.73; concordance rate 84.2–88.6%; *P*<0.001) between the manufacturer’s (IC or TC ≥25%) and 22C3 (CPS>10) algorithms (*data not shown*). Future studies should indicate which scoring algorithm is most predictive for pembrolizumab treatment selection using the SP263 assay.

The strong point of this study was its use of whole tissue sections instead of TMA punches, as this will also be used in clinical practice. One disadvantage is the relatively low number of samples, specifically of metastatic sites. The study included specimens obtained over a long time period. The median specimens’ age was, however, not significantly associated with assay discordance rate excluding differences in epitope degeneration. Finally, as in other inter-assay concordance studies, none of the patients had actually been treated with ICI, so that the most representative sampling technique and assay being predictive for therapeutic response cannot be definitely determined.

In conclusion, we observed fair to moderate agreement of PD-L1 expression outcome using the SP263 assay in matched TURBT, RC, and LN urothelial cancer samples. In matched TURBT and RC specimens, IC more often had discordant PD-L1 expression status than TC. The SP263 assay resulted in more frequent positive PD-L1 outcome than the SP142 assay, with moderate to substantial inter-assay agreement. While the SP142 assay does not include TC staining, discordant PD-L1 outcome between assays was attributed to SP263 TC staining in only 41% of cases. Based on its higher level of concordance between matched specimen types, the SP263 assay seems to represent a more robust assay for PD-L1 assessment than the SP142 test. Overall, PD-L1 expression however varied more between matched urothelial cancer specimens than between both companion assays.

## Supplementary Information

ESM 1 (DOCX 427 kb)
