# Supplementary material for: PD-L1 expression in urothelial bladder cancer varies more among specimen types than between companion assays
Source: Virchows Arch. 2021 Apr 28;479(4):705–13. doi: 10.1007/s00428-021-03094-6 (PMC8516767; doi:10.1007/s00428-021-03094-6)

**PD-L1 expression in urothelial bladder cancer varies more among specimen types than between companion assays**

Joep J. de Jong^1*^, Hans Stoop^2^, Joost L. Boormans^1^, Geert J.L.H. van Leenders^2^

^1^Department of Urology, Erasmus MC University Medical Center, Rotterdam, The Netherlands

^2^Department of Pathology, Erasmus MC University Medical Center, Rotterdam, The Netherlands

**Supplementary Tables**

**Table S1.** PD-L1 expression score of both assays

| Tissue | **SP142**  *(Positive = IC≥5%)* | **SP263**  (Positive =  *IC/TC≥25%)* | **P-value** |
| --- | --- | --- | --- |
| TURBT  Positive  Negative | 15 (15.5%)  82 (84.5%) | 38 (42.7%)  51 (57.3%) | 4x10^-5^ |
| Total | **97** | **89** |  |
| Cystectomy  Positive  Negative | 17 (17.3%)  81 (82.7%) | 39 (39.8%)  59 (60.2%) | 0.0008 |
| Total | **98** | **98** |  |
| LN+  Positive  negative | 9 (18.4%)  40 (81.6%) | 12 (27.3%)  32 (72.7%) | 0.3313 |
|  | **49** | **44** |  |

**Table S2.** Summarized Intra- and inter-agreement of the PD-L1 assays for matched TURBT, cystectomy and LN+ specimens

|  | **Concordant Cases** | **Kappa (κ)** | **Agree** | **P-value** |
| --- | --- | --- | --- | --- |
| **SP142^12^** | | | | |
| TURBT versus Cyst | 67/82 (81.7%) | 0.34 | Fair | 0.002* |
| TURBT versus LN+ | 34/44 (77.3%) | 0.04 | Slight | 0.82 |
| Cyst versus LN+ | 30/37 (81.1%) | 0.35 | Fair | 0.03* |
| **SP263** | | | | |
| TURBT versus Cyst | 59/77 (76.6%) | 0.52 | Moderate | <0.001* |
| TURBT versus LN+ | 25/36 (69.4%) | 0.31 | Fair | 0.048* |
| Cyst versus LN+ | 21/32 (65.6%) | 0.25 | Fair | 0.075 |
| **SP142 versus SP263** | | | | |
| TURBT | 66/89 (74.2%) | 0.43 | Moderate | <0.001* |
| Cyst | 73/95 (76.8%) | 0.47 | Moderate | <0.001* |
| LN+ | 35/40 (87.5%) | 0.66 | Substantial | <0.001* |

**Table S3.** Inter-assay agreement of both PD-L1 assays for matched TURBT(A), cystectomy(B) and LN+(C) specimens

1. **TURBT**

|  |  | SP142 | | |
| --- | --- | --- | --- | --- |
|  | **Score** | Negative | IC≥5% | **Total** |
| SP263 | Negative | 51 | 0 | 51 |
|  | IC≥25% | 13 | 6 | 19 |
|  | TC≥25% | 7 | 7 | 14 |
|  | IC≥25% & TC≥25% | 3 | 2 | 5 |
|  | **Total** | 74 | 15 | 89 |

**51 Concordant negative**

1. **Concordant positive**
2. **Cystectomy**

|  |  | SP142 | | |
| --- | --- | --- | --- | --- |
|  | **Score** | Negative | IC≥5% | **Total** |
| SP263 | Negative | 57 | 0 | 57 |
|  | IC≥25% | 9 | 7 | 16 |
|  | TC≥25% | 11 | 3 | 14 |
|  | IC≥25% & TC≥25% | 2 | 6 | 8 |
|  | **Total** | 79 | 16 | 95 |

**57 Concordant negative**

1. **Concordant positive**
2. **LN**

|  |  | SP142 | | |
| --- | --- | --- | --- | --- |
|  | **Score** | Negative | IC≥5% | **Total** |
| SP263 | Negative | 28 | 0 | 28 |
|  | IC≥25% | 4 | 3 | 7 |
|  | TC≥25% | 0 | 4 | 4 |
|  | IC≥25% & TC≥25% | 1 | 0 | 1 |
|  | **Total** | 33 | 7 | 40 |

**28 Concordant negative**

**7 Concordant positive**

**Supplementary Figures**

**Figure S1.** Boxplots stratifying the age of the specimens by concordant (green) and discordant (red) SP142/SP263 assay results for TURBT (A), Cystectomy (B) and LN+ (C) specimens.


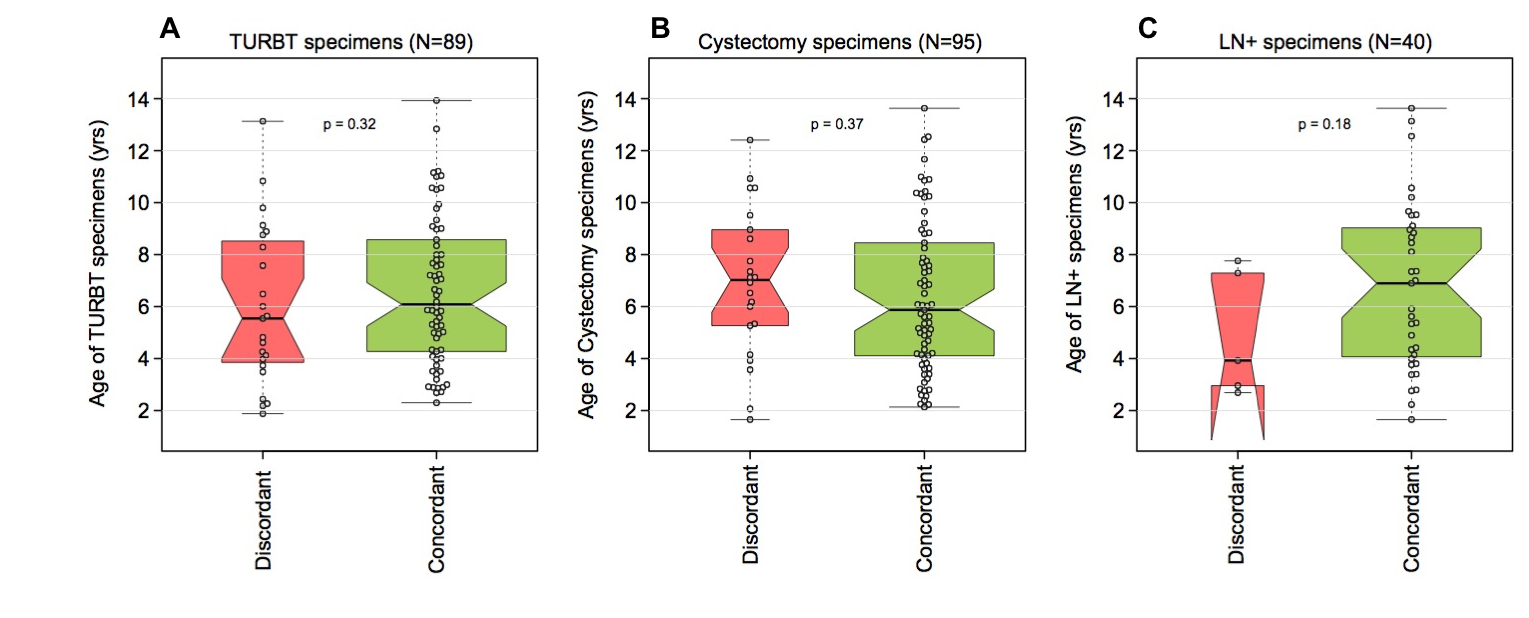

Supplement: Supplementary file 1 — (DOCX 427 kb) [file 428_2021_3094_MOESM1_ESM.docx]
